# Copper Hexacyanoferrate Thin Film Deposition and Its Application to a New Method for Diffusion Coefficient Measurement

**DOI:** 10.3390/nano11071860

**Published:** 2021-07-19

**Authors:** Jeonghun Yun, Yeongae Kim, Caitian Gao, Moobum Kim, Jae Yoon Lee, Chul-Ho Lee, Tae-Hyun Bae, Seok Woo Lee

**Affiliations:** 1 School of Electrical and Electronic Engineering, Nanyang Technological University, Singapore 639798, Singapore; jhyun@ntu.edu.sg (J.Y.); y.kim@ntu.edu.sg (Y.K.); ctgao@hnu.edu.cn (C.G.); moobum001@e.ntu.edu.sg (M.K.); jylee89@korea.ac.kr (J.Y.L.); 2 KU-KIST Graduate School of Converging Science and Technology, Korea University, Seoul 02841, Korea; chlee80@korea.ac.kr; 3 Department of Chemical and Biomolecular Engineering, Korea Advanced Institute of Science and Technology, Daejeon 34141, Korea; thbae@kaist.ac.kr

**Keywords:** prussian blue analogue, thin film, micropattern, diffusion coefficient

## Abstract

The use of Prussian blue analogues (PBA) materials in electrochemical energy storage and harvesting has gained much interest, necessitating the further clarification of their electrochemical characteristics. However, there is no well-defined technique for manufacturing PBA-based microelectrochemical devices because the PBA film deposition method has not been well studied. In this study, we developed the following deposition method for growing copper hexacyanoferrate (CuHCFe) thin film: copper thin film is immersed into a potassium hexacyanoferrate solution, following which the redox reaction induces the spontaneous deposition of CuHCFe thin film on the copper thin film. The film grown via this method showed compatibility with conventional photolithography processes, and the micropattern of the CuHCFe thin film was successfully defined by a lift-off process. A microelectrochemical device based on the CuHCFe thin film was fabricated via micropatterning, and the sodium ion diffusivity in CuHCFe was measured. The presented thin film deposition method can deposit PBAs on any surface, including insulating substrates, and it can extend the utilization of PBA thin films to various applications.

## 1. Introduction

Prussian blue analogues (PBAs) are characterized by a rigid open framework and consist of transition metals and cyano-bridges. They have been utilized in various applications such as electrochemical sensing [1,2], energy storage [3,4], and energy harvesting [5]. Among them, the energy storage application has aroused the most attention because of the stable cyclic performance and rate capability of PBAs [6]. The energy loss of PBA-based batteries throughout their charge–discharge cycle is lower than that of conventional battery materials [7,8], and the batteries have fast charging and discharging rates [9]. Because PBAs can function in the presence of various alkaline ions and even multivalent ions, various ion-based batteries have been developed [10]. In addition, PBA-based batteries can harvest low-grade heat energy because of their high energy efficiency [5,11,12].

There are several methods for synthesizing PBAs. The coprecipitation method is one of the most widely used methods for synthesizing PBA powder because it allows mass production at low cost [13]. Additives such as citrate and polyvinylpyrrolidone have been introduced into precursors to improve the specific capacity and cyclic performance [14,15]. Methods based on the use of a single-salt solution have been employed to obtain Prussian blue with low vacancy and water amount, which enhances the specific capacity and cyclic performances [16,17]. Besides bulk synthesis, several other methods have been reported for depositing PBA films. Electrodeposition methods have been studied to deposit a Prussian blue thin film on electrodes, which were then utilized as electrochemical sensors and electrochromic devices [18,19,20]. Metal hexacyanoferrate films have been deposited by applying cyclic potential [21,22]. However, the PBA films are only deposited on conductive electrodes subjected to electric potential. To overcome this limitation, a method of growing PBA films by repeatedly immersing the substrate into two precursors has been demonstrated [23,24]. Unlike the electrodeposition methods, this immersion method can deposit PBA films on any surface regardless of the surface electrical conductivity; however, when the number of immersion repetitions is increased, the PBA films grow as particle-like structures rather than a conformal film. Well-developed PBA coating methods can expand the utilization of PBAs with superior physical and electrochemical properties to various applications, from batteries to microscale devices integrated with PBA thin films.

In this paper, we introduce a new method to obtain a thin film of copper hexacyanoferrate (CuHCFe) by dipping a copper metal film into a potassium hexacyanoferrate solution. The copper film is oxidized by reducing hexacyanoferrate, and the copper ion subsequently forms CuHCFe by reacting with the reduced hexacyanoferrate. The temperature and concentration of hexacyanoferrate solutions influenced the surface morphology of the CuHCFe thin film. The CuHCFe thin film was characterized via electrochemical and spectroscopic methods. Micropatterning of the CuHCFe thin film through the lift-off process was used to develop a PBA-based microelectrochemical system to measure the electrical current of the CuHCFe thin film during electrochemical reaction. The diffusivity of sodium ions in CuHCFe was estimated using a numerical model of the electrical current.

## 2. Materials and Methods

### 2.1. Materials

Sodium chloride (NaCl), potassium chloride (KCl), and potassium hexacyanoferrate (K_3_Fe(CN)_6_) were purchased from Sigma-Aldrich. The AZ 5214e photoresist (PR) and an AZ developer were purchased from Microchemical. Acetone and isopropanol were purchased from J.T.Baker (Phillipsburg, NJ, USA), and deionized (DI) water was obtained from Direct-Q 3 UV water purification system.

### 2.2. Deposition of CuHCFe Thin Film

Copper metal thin film was deposited by a physical vapor deposition method of e-beam evaporation. The 20 nm thick of copper thin film was deposited on a platinum deposited glass substrate. The substrate was dipped into 1 mM K_3_Fe(CN)_6_ solution for 24 h at room temperature (25 °C) to form the CuHCFe thin film. The coated substrate was rinsed with DI water and dried at 40 °C for a day in a convection oven.

### 2.3. Micropatterning of CuHCFe Thin Film

The micropattern of CuHCFe thin film was obtained through a lift-off process. First, various patterns of the PR were defined on a platinum-coated glass substrate via photolithography. Then, 20 nm–thick copper thin film was deposited on the substrate through e-beam evaporation. The copper coated substrate was dipped into 1 mM K_3_Fe(CN)_6_ solution for 24 h at room temperature (25 °C) and then rinsed with DI water. The PR was removed using acetone, and the coated substrate was rinsed with isopropanol and DI water and dried at 40 °C for a day in a convection oven.

### 2.4. Electrochemical Characterization of CuHCFe Thin Films

The cyclic voltammetry analyses of the CuHCFe thin film and the micropatterned CuHCFe thin film were conducted using a potentiostat/galvanostat (SP300, Biologic, Seyssinet-Pariset, France). A three-electrode beaker cell was prepared with a platinum electrode coated with the CuHCFe thin film (or the micropatterned CuHCFe thin film) as the working electrode, activated carbon-coated carbon cloth as the counter electrode, and Ag/AgCl filled with saturated KCl electrolyte as the reference electrode. The electrodes were immersed in 1 M NaCl (or 1 M KCl) aqueous electrolyte. The potential scan range was 0.4 to 0.9 V (vs. Ag/AgCl), with a scan rate of 1 mV/s. To compare the electrochemical behaviors of the CuHCFe thin film and CuHCFe powder synthesized via a coprecipitation method, a conventional CuHCFe electrode and the CuHCFe powder were analyzed via cyclic voltammetry. All electrochemical measurements were conducted at 25 °C in atmosphere.

### 2.5. Characterization of CuHCFe Thin Film

Atomic force microscopy (AFM; Dimension Edge, Bruker, Billerica, MA, USA) was performed to measure the surface morphology of the CuHCFe thin films. Scanning electron microscopy (SEM; Apreo 2, Thermo Fisher Scientific, Waltham, MA, USA) was performed to determine the morphology of the CuHCFe thin films. The ultraviolet–visible (UV–vis) spectra of the K_3_Fe(CN)_6_ solutions before and after reaction with copper were obtained using the Shimadzu UV 2450 spectrometer. The solutions for UV–vis analysis were contained in disposable cuvettes (UV-cuvette semi-micro, Brand, Wertheim, Germany). The Raman spectrum of the CuHCFe thin film was obtained using a lab-made system with laser (532 nm) and Raman spectrometer (UHTS 300, WITec, Ulm, Germany) at −70 °C.

## 3. Results and Discussion

### 3.1. CuHCFe Thin Film Deposition

**Figure 1:**
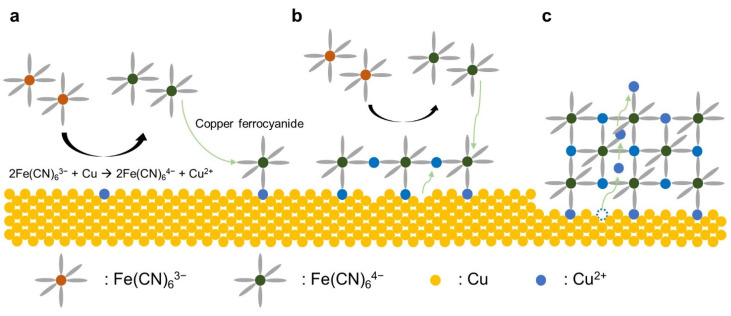
Schematic of a CuHCFe thin film deposition by corrosion of copper metal thin film in a K_3_Fe(CN)_6_ solution: (**a**) copper oxidation and copper ferrocyanide formation, (**b**) thin film formation, and (**c**) growth of CuHCFe film.

Figure 1 shows the schematic of the growth mechanism of the CuHCFe thin film, starting from the immersion of a copper metal thin film into the K_3_Fe(CN)_6_ solution. The corrosion reaction of copper with ferricyanide (Fe(CN)_6_^3−^) occurred at the interface of the metal thin film and the solution (Figure 1a). The oxidized copper simultaneously reacted with ferrocyanide (Fe(CN)_6_^4−^), resulting in the formation of copper ferrocyanide at the interface. Because the reaction between transition metals and ferrocyanide is very fast, the oxidized copper formed copper ferrocyanide, which attached to the surface instead of floating in the liquid [25]. By further oxidizing copper, the surface of the copper film is covered by CuHCFe (Figure 1b). The copper metal thin film underneath the CuHCFe thin film was continuously oxidized by the continuous reduction of Fe(CN)_6_^3−^ and the copper ions are transferred to the top of the CuHCFe film (Figure 1c). We believe that the copper ions were transferred through the CuHCFe thin film because the copper ions can intercalate into and deintercalate from CuHCFe [26]. Therefore, copper was oxidized and then intercalated into the CuHCFe at the bottom of CuHCFe because oxidation potential of Cu/Cu^2+^ is lower than formal potential of CuHCFe with intercalating copper ion. Copper ions deintercalated from the top surface of CuHCFe film and reacted with Fe(CN)_6_^4−^, forming CuHCFe. The CuHCFe thin film deposition reaction continued until the copper was fully oxidized.

**Figure 2:**
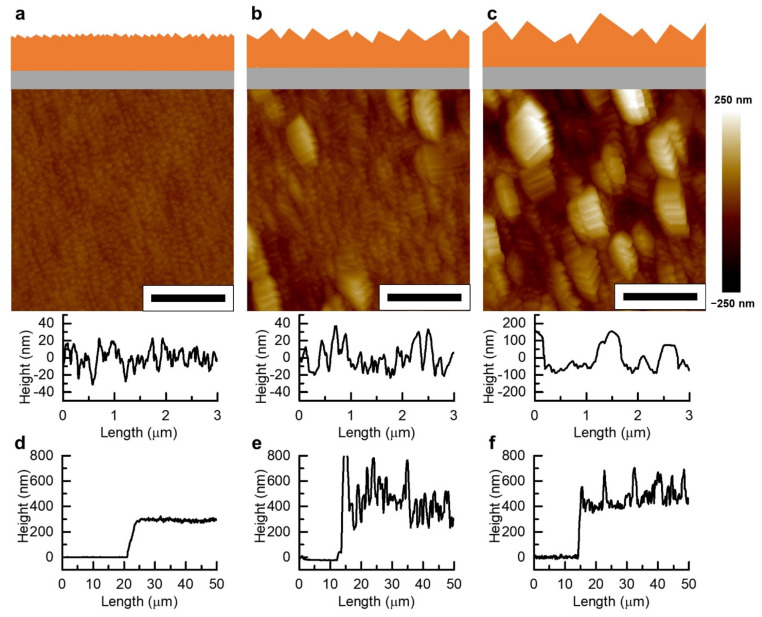
AFM images of CuHCFe thin films grown at (**a**) 5, (**b**) 25, and (**c**) 60 °C. Line profiles of the step edge of CuHCFe thin film grown at (**d**) 5, (**e**) 25, and (**f**) 60 °C. Scale bar: 1 μm.

The surface morphologies of the CuHCFe thin films grown at different temperatures were characterized via AFM (Figure 2). The morphology of the CuHCFe thin film grown at high temperature (60 °C) was rougher than that of the film grown at low temperature (5 °C). The higher temperature induced the faster oxidation of copper metal and the transportation of copper ion through the CuHCFe thin film, resulting in a larger CuHCFe nanocrystal. Likewise, the lower temperature induced slower oxidation rates, resulting in the formation of smaller CuHCFe nanocrystals and smoother surface morphology. The surface roughnesses of the CuHCFe thin films were calculated based on the AFM images using a software program. The roughnesses (Ra) of the CuHCFe thin films grown at 5, 25, and 60 °C were 7.44, 20.5, and 61.8 nm, respectively. The line profiles of the CuHCFe thin films also showed that the height variation of the CuHCFe thin film grown at 5 °C was smaller than those of the CuHCFe thin films grown at 25 and 60 °C.

The line profiles of the step edge of CuHCFe thin films were characterized via AFM (Figure 2e,f). The thicknesses of the CuHCFe thin films grown at 5, 25, and 60 °C were 292, 464, and 481 nm, respectively. The CuHCFe thin film was much thicker than the initial copper metal thin film (20 nm) because of a 2133% volumetric expansion in the thin film lattice during transformation. The lattice structures of copper metal and CuHCFe are face-centered cubic (FCC, space group Fm-3 m), and the lattice parameters of copper metal and CuHCFe are 0.362 and 1.004 nm, respectively [7,27]. In the CuHCFe thin film, the film can expand in the direction normal to the copper surface because there is no lateral expansion of film. Therefore, the linear expansion of thickness during transformation from the copper thin film to a CuHCFe thin film was also 2133%, with the theoretical thickness of the CuHCFe thin film being 426.6 nm. The thicknesses of the CuHCFe thin films grown at 25 and 60 °C were greater than the theoretical thickness. The large crystalline structures of the CuHCFe thin films grown at 25 and 60 °C resulted in inaccuracies in the thickness measurement. However, the thickness of the CuHCFe thin film grown at 5 °C was lower than the theoretical value. We believe that unreacted copper metal film remained underneath the CuHCFe thin film because of the slow transportation of copper ions in the CuHCFe thin film at 5 °C. Although we prolonged the immersion time of the copper metal film in the potassium ferricyanide solution to 96 h, the thickness of the CuHCFe thin film was 250 nm, which was still lower than the theoretical thickness. Thus, the temperature of 25 °C or above was required to fully transform CuHCFe from copper. In addition, mass analysis proved the transformation of CuHCFe film from copper metal film. (See details for Supporting Information).

**Figure 3:**
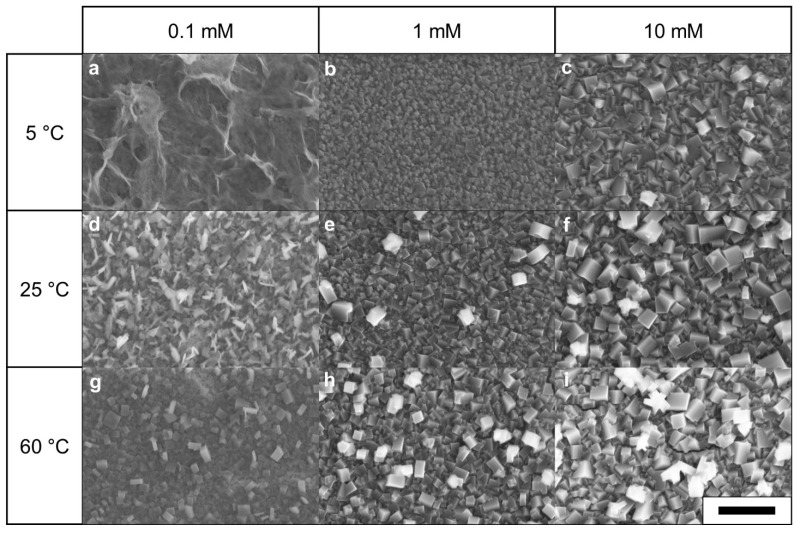
SEM images of CuHCFe thin films grown at various temperatures and concentrations of K_3_Fe(CN)_6_ solution: (**a**) 0.1 mM & 5 °C, (**b**) 1 mM & 5 °C, (**c**) 10 mM & 5 °C, (**d**) 0.1 mM & 25 °C, (**e**) 1 mM & 25 °C, (**f**) 10 mM & 25 °C, (**g**) 0.1 mM & 60 °C, (**h**) 1 mM & 60 °C, and (**i**) 10 mM & 60 °C. Scale bar: 1 μm.

Figure 3 shows that the K_3_Fe(CN)_6_ solution temperature (5, 25, and 60 °C) and concentration (0.1, 1, and 10 mM) influenced the CuHCFe thin film surface morphology. Consistent with the AFM results, the SEM images showed that the CuHCFe thin film surface was rough at the high solution temperature. As the temperature and concentration of the K_3_Fe(CN)_6_ solution increased, so did the crystal size of CuHCFe. At 60 °C and in 10 mM K_3_Fe(CN)_6_ solution, owing to the fast reaction, the CuHCFe structure featured particle-like deposition rather than a thin film layer. The CuHCFe thin film grown at 5 °C and 0.1 mM of K_3_Fe(CN)_6_ was not crystalline because of the low ferrocyanide flux toward the surface. Under high ferrocyanide flux toward the surface, the oxidized copper immediately reacted with ferrocyanide on the CuHCFe surface such that the CuHCFe structure became more crystalline. In contrast, under low ferrocyanide flux, copper ions diffused into the solution and then reacted with ferrocyanide. Figure 3g shows that the CuHCFe thin film grown at 60 °C and 0.1 mM K_3_Fe(CN)_6_ had a crystalline CuHCFe structure because of the high diffusivity of ferrocyanide at the high temperature despite the low solution concentration. Based on the morphology analysis by AFM and SEM, the CuHCFe thin film grown at 25 °C and 1 mM K_3_Fe(CN)_6_ was selected for the rest of the experiment because the copper thin film was fully transformed and the grown CuHCFe film was not too rough.

**Figure 4:**
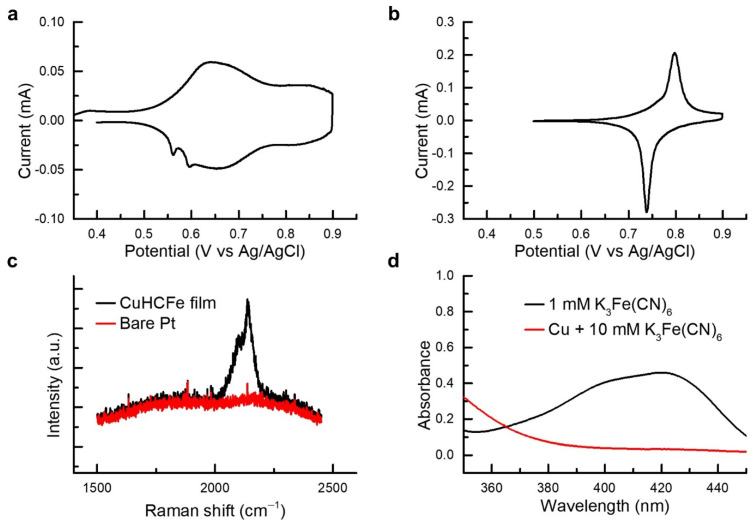
Cyclic voltammetry curves of the CuHCFe thin film in (**a**) 1 M NaCl and (**b**) 1 M KCl. (**c**) Raman spectroscopy of the CuHCFe thin film. (**d**) UV-vis spectroscopy of the reacted K_3_Fe(CN)_6_ solution.

The electrochemical property of the CuHCFe thin film was characterized via cyclic voltammetry in 1 M NaCl and 1 M KCl aqueous electrolytes (Figure 4a,b). The 1 cm × 1 cm CuHCFe thin film was deposited on a platinum thin-film electrode. The peak potentials of cyclic voltammetry curves at 0.642 and 0.798 V (vs. Ag/AgCl) for 1 M NaCl and 1 M KCl electrolytes, respectively, indicate the redox reaction of the CuHCFe thin film. As shown in Figure S1, the cyclic voltammetry curve of the CuHCFe thin film matched well with that of CuHCFe powder synthesized via coprecipitation method. The open-circuit voltage of the CuHCFe thin film after deposition was 0.322 V (vs. Ag/AgCl) in 1 M NaCl, which corresponds to the reduced state of CuHCFe. This means that the CuHCFe thin film was formed from the reaction of copper ions with Fe(CN)_6_^4−^ ions rather than Fe(CN)_6_^3−^ ions. Furthermore, the CuHCFe thin film was analyzed via Raman spectroscopy (Figure 4c). The peaks at 2140 and 2100 cm^−1^ are attributed to the vibration mode of cyano-bond from CuHCFe intercalated with potassium ions [28]. The cyclic voltammetry and Raman spectroscopy results verify that the copper thin film was successfully converted to CuHCFe thin film.

The K_3_Fe(CN)_6_ solution after reaction with copper metal was analyzed via UV–vis spectroscopy. Copper powder (10 mmol) was dispersed into 100 mL of 10 mM K_3_Fe(CN)_6_ solution for 40 h with vigorous stirring. The molar amount of copper powder was 10 times that of K_3_Fe(CN)_6_ to enable the complete reaction of the K_3_Fe(CN)_6_ solution. The reacted K_3_Fe(CN)_6_ solution was collected using a centrifuge, and its absorbance was measured. The spectrum of the reacted solution showed the absence of ferricyanide ions (Figure 4d), which indicates that the K_3_Fe(CN)_6_ solution was reduced by copper metal and proves that copper was oxidized via Fe(CN)_6_^3−^ reduction, as explained in the proposed mechanism of CuHCFe thin film deposition.

### 3.2. Micropatterning of CuHCFe Thin Film and Fabrication of Microelectrochemical System of CuHCFe

The CuHCFe thin film deposition method can be utilized to fabricate a CuHCFe-based microelectrochemical device. Because this deposition method can deposit CuHCFe thin film on a nonconductive substrate, CuHCFe thin film can be deposited between two separated metal electrodes. Given that the conventional electrodeposition method requires subjecting the electrode to electric potential, it cannot realize deposition on a nonconductive substrate. Our deposition method allows the deposition of a uniform CuHCFe thin film between two separated electrodes regardless of the gap width between two electrodes.

**Figure 5:**
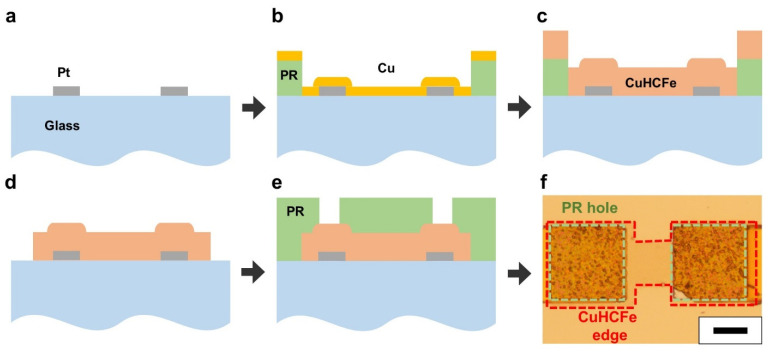
Fabrication process of CuHCFe-based microelectrochemical device: (**a**) Pt electrode patterning, (**b**) photolithography and copper deposition, (**c**) formation of CuHCFe thin film by immersing into a K_3_Fe(CN)_6_ solution, (**d**) removing PR by acetone, and (**e**) passivation by photolithography. (**f**) Optical microscope image of the device. Scale bar: 20 μm.

The microelectrochemical device was fabricated through conventional microfabrication processes and the CuHCFe thin film deposition. First, platinum electrodes were defined on a glass substrate via physical vapor deposition and a lift-off process (Figure 5a). Then, the PR AZ5214E was patterned via photolithography, and the copper metal thin film was deposited via physical vapor deposition (Figure 5b). The CuHCFe thin film was obtained by immersing the substrate into a 1 mM K_3_Fe(CN)_6_ solution for 24 h at 25 °C (Figure 5c). The coated substrate was rinsed with DI water and dried via nitrogen blowing. The PR was removed using acetone and rinsed with isopropanol and DI water. The substrate was dried in a convection oven at 40 °C for a day (Figure 5d). To prevent electrical leakage through the electrolyte onto the platinum electrodes and the CuHCFe thin film, their surfaces were covered with PR except for the area where the CuHCFe thin film and platinum electrode were stacked (Figure 5e). The optical microscopy image of the CuHCFe microdevice shows that the layers of platinum electrodes, CuHCFe thin film, and PR were well stacked (Figure 5f).

As described above, the CuHCFe thin film could be micropatterned through photolithography and the lift-off of the CuHCFe thin film. The methods enabled the deposition of the desired size of CuHCFe thin film on the desired area. We deposited an array of microsized CuHCFe thin film on a platinum electrode to characterize the microsized CuHCFe thin film. Dot and line patterns of the CuHCFe thin film were deposited. The optical microscope image of the microsized CuHCFe thin film (Figure S2a–c) shows that CuHCFe thin films of any size can be deposited. The cyclic voltammetry of the microsized CuHCFe thin film (Figure S2d) shows that conventional photolithography did not affect the electrochemical behavior of the CuHCFe thin film.

**Figure 6:**
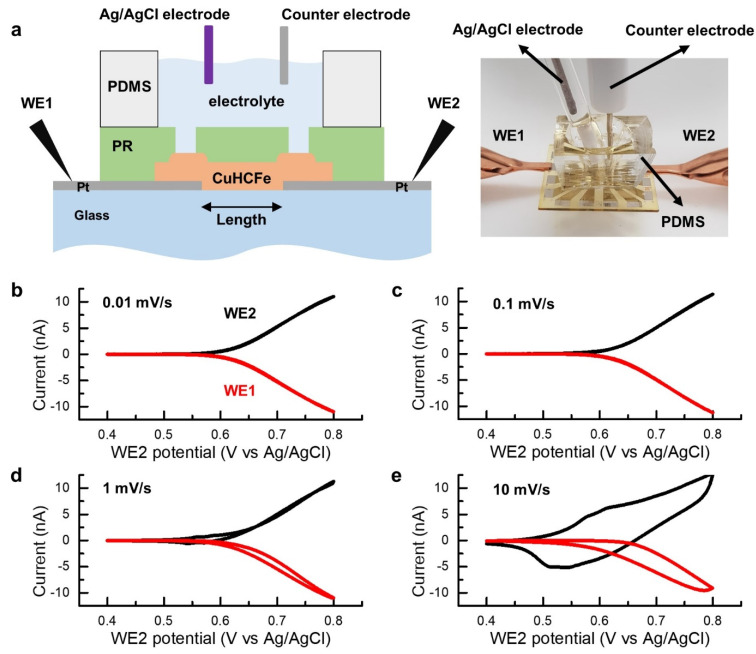
(**a**) Electrochemical system with the microdevice. 0.4 V (vs. Ag/AgCl) was applied to WE1 and sweeping potential between 0.4 and 0.8 V (vs. Ag/AgCl) was applied to WE2. The electrical current of WE1 and WE2 at different scan rate of WE2 ((**b**): 0.01 mV/s, (**c**): 0.1 mV/s, (**d**): 1 mV/s, and (**e**): 10 mV/s).

Furthermore, a CuHCFe-based microelectrochemical device was fabricated and used to measure the electrical conductance of the micropatterned CuHCFe thin film (Figure 6a). A polydimethylsiloxane (PDMS) well was assembled with the CuHCFe microdevice and filled with an aqueous electrolyte of 1 M NaCl. A Ag/AgCl electrode in saturated KCl solution and a platinum wire were used as the reference electrode and counter electrode, respectively (Figure 6a). Bipotentiostat mode of potentiostat (SP300, BioLogic) was used to apply the working potential to two platinum electrodes. In the bipotentiostat mode, two working electrodes are sharing the reference electrode and the counter electrode and the potential of each working electrode can be controlled independently and the relevant current measured. The dimensions of the CuHCFe thin film between two electrodes were 20 μm (width) × 10 μm (length) × and 426 nm (thickness).

Constant potential was applied to one electrode (working electrode 1, WE1) and cyclic potential was applied to the other electrode (working electrode 2, WE2). Figure 6b–e show the measured currents of WE1 and WE2 when 0.4 V (vs. Ag/AgCl) was applied to WE1 and cyclic potentials from 0.4 to 0.8 V (vs. Ag/AgCl) at the scan rates of 0.01, 0.1, 1, and 10 mV/s were applied to WE2. Two types of currents were generated in this electrochemical cell: current flowing through the CuHCFe thin film because of the electrical conductivity of CuHCFe and the potential difference between WE1 and WE2 and redox current from the redox reaction of the CuHCFe thin film on a Pt electrode. The electronic conductivity of intercalation materials depends on the cation concentration gradient in the materials [29]. From the cyclic voltammetry result of the CuHCFe thin film (Figure 4a), we infer that sodium ions started to deintercalate at 0.55 V (vs. Ag/AgCl). Therefore, when the WE2 potential was lower than 0.55 V (vs. Ag/AgCl), the conductivity of the CuHCFe thin film was low because of the small gradient of sodium ion concentration in the CuHCFe film. The conductivity gradually increased as the potential of WE2 increased, because the cation concentration gradient between WE1 and WE2 increased with increasing WE2 potential. Hence, the CuHCFe thin film exhibited a nonlinear current behavior (Figure 6b). Besides the electronic current through CuHCFe film, the ionic current is minor compared to the electronic current and zero at the steady state. Because a constant potential was applied to WE1, there was no redox reaction of the CuHCFe on WE1, which means that the current in WE1 did not contain redox current. However, because of the cyclic potential applied to the electrode, the current in WE2 contained redox current from the CuHCFe on WE2. Consequently, the current of WE2 comprised the electronic current through the CuHCFe thin film and the current from the redox reaction of the CuHCFe on a Pt electrode. The current of WE1 was only that flowing through the CuHCFe thin film from WE1 to WE2. As the scan rate increased, the current hysteresis on WE1 increased, and the redox current on WE2 increased. The hysteresis came from the slow diffusion of cation in CuHCFe. The increase in redox current occurred because the peak current of cyclic voltammetry is proportional to the scan rate to the power of b (b = 0.5–1) [30].

**Figure 7:**
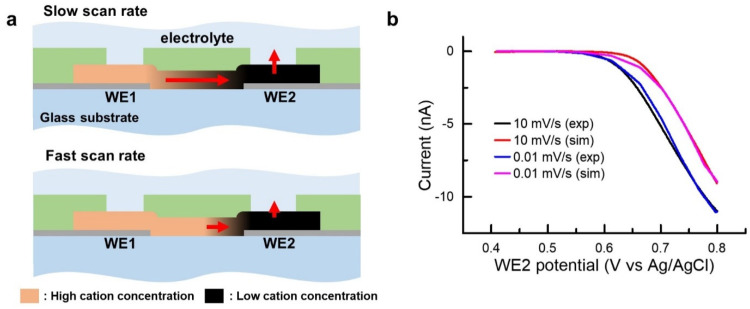
(**a**) Schematic of cation concentration in CuHCFe film under forward scanning of WE2. (**b**) Experimental and simulated currents of WE1 at different scan rates of WE2 (0.01 and 10 mV/s).

The current hysteresis of the CuHCFe thin film at a high scan rate implies that the cation diffusivity in CuHCFe could affect the current. Herein, we developed a model to investigate the effect of cation diffusion on the electronic current in the CuHCFe thin-film device. Figure 7a shows the electrochemical state of the CuHCFe film at fast and slow scan rates. At the fast scan rate, there is not enough time for sodium ions inside the CuHCFe thin film to diffuse out. Sharp cation concentration gradient in the CuHCFe film is built near WE2 and gradual concentration gradient is built near WE1. Because the electrical conductivity of intercalation materials is proportional to ion concentration in the materials, the electrical conductivity of the CuHCFe film is low due to the gradual concentration gradient near WE1. Figure 7b shows the WE1 current under forward scanning at rates of 0.01 and 10 mV/s and the current calculated by a numerical simulation. We investigated the currents of WE1 at different scan rates because the current of WE1 can be assumed to be the electronic current through the CuHCFe thin film. The WE1 current at the scan rate of 10 mV/s showed a delayed response compared with that at 0.01 mV/s. This delayed response resulted from the relatively slow transportation of sodium ions in the CuHCFe thin film. Through the finite element method, the diffusivity of sodium ions in the CuHCFe film was estimated. The detailed method is described below.

The simplified equation for determining the concentration of intercalated cation in CuHCFe is defined below:

$$C\left(E\right)=C_{0}\left(\frac{1}{1+e^{f\left(V\right)}}\right)$$

where C_0_ is the maximum concentration of cation in CuHCFe, and f(V) is a quintic function. In this study, f(V) was obtained by fitting the galvanostatic charge of the CuHCFe film, and C_0_ was calculated based on the dimensions and capacity of CuHCFe (Figure S3, Supporting Information). Cation transportation in the CuHCFe thin film was driven by Fick’s second law of diffusion. The cation concentration inside the CuHCFe thin film at the fast scan rate was higher than that at a steady state. The excess cation was diffused. Fick’s second law of diffusion for cation is described below:

$$\frac{\partial C}{\partial t}=D\frac{\partial^{2}\left(C-C\left(E\right)\right)}{\partial x^{2}}$$

where D is the diffusion constant of cation in CuHCFe, C is the cation concentration in CuHCFe, and x is the length from WE1. The difference between the cation concentration (C) and the intercalated cation concentration (C(E)) can be built while sweeping WE2 potential. We assume that the electrical conductivity (σ) of CuHCFe is proportional to the absolute of the gradient of cation concentration [29]. The electrical current density of CuHCFe is described as follows:

$$J\left(x\right)=\sigma \frac{\partial E}{\partial x}=\left(\sigma_{0}\left|\frac{\partial C}{\partial x}\right|\right)\left(\frac{\partial E}{\partial x}\right)$$

where σ_0_ is a constant for conductivity. The electrical potential at x = 0 μm (E_x=0_) is 0.4 V, and the electrical potential at x = 10 μm (E_x=10_) is swept from 0.4 to 0.8 V at the rate of 0.01 and 10 mV/s. We assume that the cation diffusion between the CuHCFe thin film and electrolyte is faster than that inside the CuHCFe thin film. Thus, the cation concentrations at x = 0 and x = 10 μm are C(E_x=0_) and C(E_x=10_), respectively. At the scan rate of 0.01 mV/s, the electrical current curves of forward scanning and backward scanning were identical, which means that the cation concentration had reached a steady state (C=C(E)). Thus, σ_0_ was calculated by curve fitting with the current at 0.01 mV/s, and D was calculated by curve fitting with the current at 10 mV/s. As shown in Figure 7, the electrical current fit well with σ_0_ × C_0_ of 5.77 × 10^−7^ S and D of 8.8 × 10^−9^ cm^2^/s (Supporting Information, Figure S4, σ along the *x*-axis) The calculated diffusivity was comparable with that of the previously reported values measured by the galvanostatic intermittent titration technique and electrochemical impedance spectroscopy [31,32]. We believe that the microelectrochemical system can be utilized as an alternative method to measure the diffusivity of cation in intercalation materials using the above approach.

## 4. Conclusions

In summary, we developed a CuHCFe thin film growth process involving the immersion of a copper metal thin film into a potassium hexacyanoferrate solution. The surface morphologies of CuHCFe thin films grown at different conditions (temperature and concentration of potassium hexacyanoferrate solution) were characterized via AFM and SEM. The CuHCFe thin film grown at 5 °C had smooth surface morphology; however, the 20 nm–thick copper metal film was not fully converted to CuHCFe thin film. The CuHCFe thin film grown at 25 °C was characterized via cyclic voltammetry and Raman spectrometry. Because the grown film is compatible with the conventional photolithography processes, a micropattern array of the CuHCFe thin film was deposited at a desired location using the lift-off process. A CuHCFe-based microelectrochemical device was fabricated, and an electrochemical system was built to measure the electrical current of the CuHCFe thin film. The diffusivity of sodium ions in the CuHCFe thin film was calculated through the numerical simulation of electrical conduction and diffusion of the CuHCFe thin film. The proposed film deposition method can be applied for the deposition of not only CuHCFe but also PBAs because the method is not limited to copper metal; it can also be applied for nonconductive substrates and arbitrary surfaces. The CuHCFe microdevice paves the way for new types of electrochemical transistors and sensors and provides a means to comprehensively clarify PBAs.

## Data Availability

The data presented in this study are available on request from the corresponding author.

## Supplementary Materials

The following are available online at https://www.mdpi.com/article/10.3390/nano11071860/s1, Figure S1: cyclic voltammetry curves of the CuHCFe thin film and CuHCFe powder synthesized by co-precipitation, Figure S2: Optical microscope images of patterned CuHCFe film, Figure S3: Specific capacity of a CuHCFe thin film in 1 M NaCl electrolyte and fitted curve, Figure S4: Calculated conductivity along *x*-axis.
