# Supplementary material for: Copper Hexacyanoferrate Thin Film Deposition and Its Application to a New Method for Diffusion Coefficient Measurement
Source: Nanomaterials (Basel). 2021 Jul 19;11(7):1860. doi: 10.3390/nano11071860 (PMC8308250; doi:10.3390/nano11071860)
Supplement: Supplementary file 1 [file nanomaterials-11-01860-s001.zip › nanomaterials-1283247-SI.pdf]

Supplementary Materials

Copper hexacyanoferrate thin film deposition and its characterization in micro-electrochemical device

Jeonghun Yun [1], Yeongae Kim [1], Caitian Gao [1], Moobum Kim [1], Jae Yoon Lee [1,2], Chul-Ho Lee [2], Tae-Hyun Bae [3] and Seok Woo Lee [1,*]

[1] School of Electrical and Electronic Engineering, Nanyang Technological University, Singapore, 639798, Singapore
[2] KU-KIST Graduate School of Converging Science and Technology, Korea University, Seoul 02841, Republic of Korea
[3] Department of Chemical and Biomolecular Engineering, Korea Advanced Institute of Science and Technology, Daejeon, 34141, Republic of Korea
* Correspondence: sw.lee@ntu.edu.sg

Mass analysis

We measured the mass change upon the transformation from copper to CuHCFe using microbalance (XP26, Mettler Toledo). There was 106 μg increment when 2 cm (width) × 1 cm (length) × and 20 nm (thickness) of copper film was dipped into a ferricyanide solution. From the dimension, the mass of copper film is 35.84 μg and the mole of copper film is 0.564 μmol. The molecular weight of hexacyanoferrate ($Fe(CN)_6$) is 211.95 g/mol. Theoretical weight increment after transforming is 119.5 μg (0.564 μmol × 211.95 g/mol). We think that the different between measured and theoretical values comes from measurement error and incorrect dimension of film. The actual copper film should be smaller than 2 cm (width) × 1 cm (length) because we cut the copper deposited glass using a blading saw (ADT 7120, Advanced Dicing Technologies). The mass analysis verifies the transforming CuHCFe film from copper.

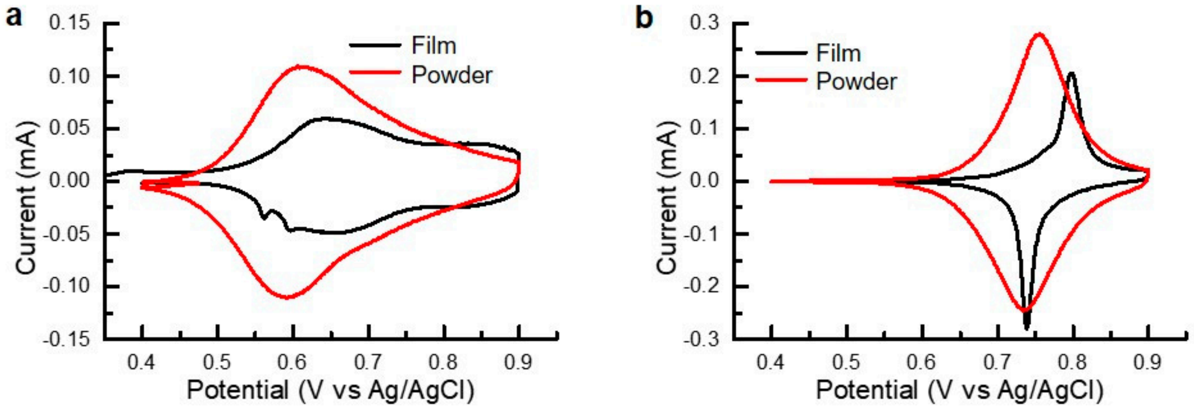

Figure S1. CV curves of the CuHCFe thin film and CuHCFe powder synthesized by co-precipitation: (a) 1 M NaCl and (b) 1 M KCl.

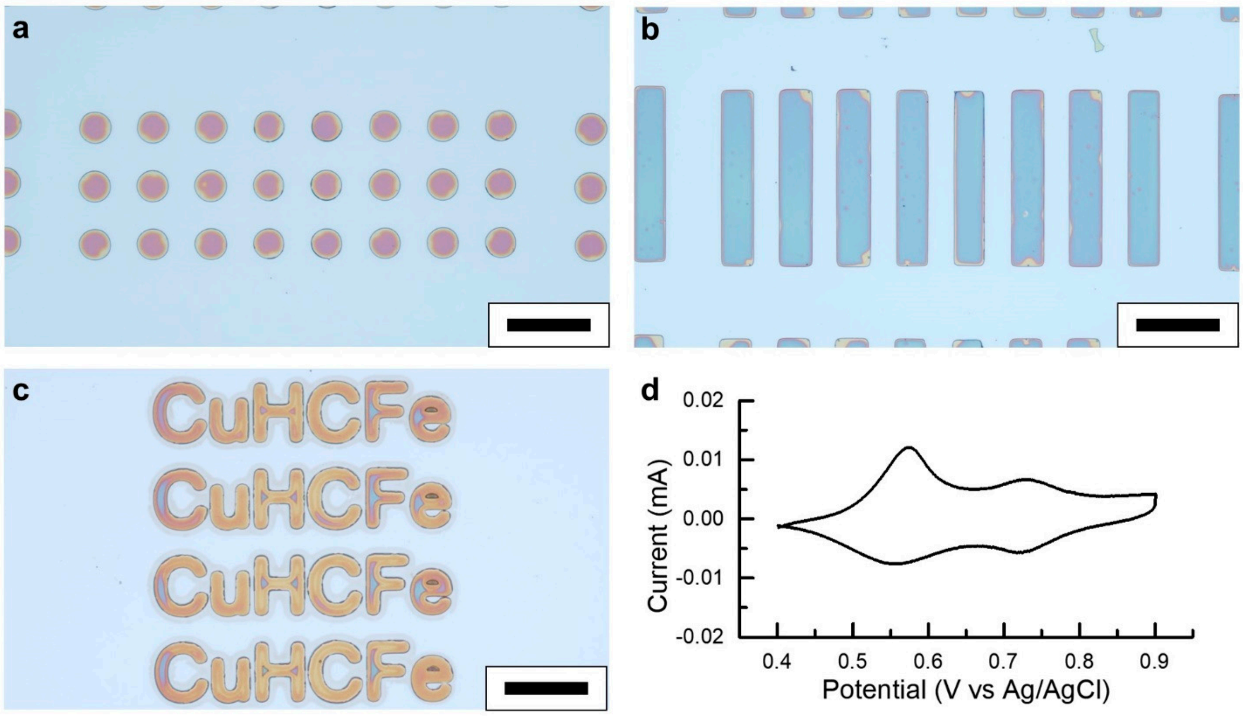

Figure S2. Optical microscope images of patterned CuHCFe film: (a) dot, (b) line, and (c) letter of CuHCFe. Scale bar: 100 µm. (d) CV curve of dot-patterned CuHCFe film in 1 M NaCl.

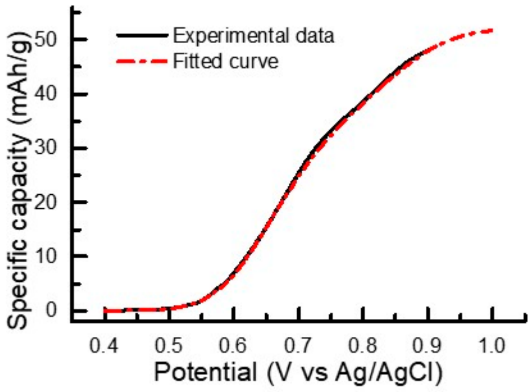

Figure S3. Specific capacity of a CuHCFe thin film in 1 M NaCl electrolyte and fitted curve. f(V) was a polynomial of degree 5 because polynomials of degree up to 4 were unable to represent the data at the end of the graph well. The error caused large difference of calculated current. In here, f(V) is -574.69*V^5+2273.8*V^4-3407.3*V^3+2404.3*V^2-773.67*V+82.72.

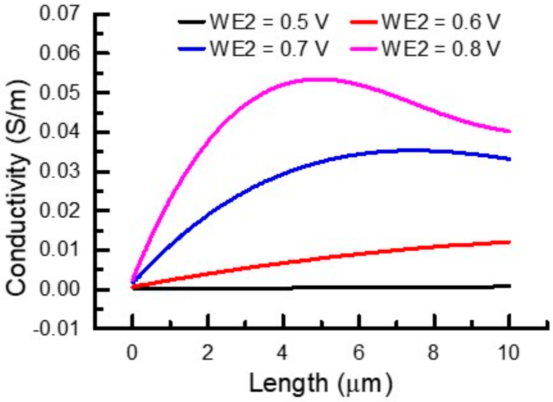

Figure S4. Calculated conductivity ($\sigma_0 \cdot |\partial C/\partial x|$) along x-axis.
